# Eversion cruroplasty and collar overwrap: a novel hybrid approach for refractory gastroesophageal reflux disease in children, with assessment of mid-term outcomes

**DOI:** 10.1007/s00464-024-11448-9

**Published:** 2025-01-06

**Authors:** Hamed M. Seleim

**Affiliations:** Faculty of Medicine, Pediatric Surgery, Tanta University Hospital, Tanta, 31527 Egypt

**Keywords:** Pediatric GERD, Cruroplasty, Hybrid technique, Partial wrap, Belching, Dysphagia

## Abstract

**Background:**

Surgical fundoplication remains integral in managing gastroesophageal reflux disease (GERD) by addressing gastroesophageal valve incompetence. This study introduces a novel hybrid approach, the Eversion Cruroplasty and Collar Overwrap (ECCO) procedure, aiming to combine benefits of conventional partial wrapping and posteromedial cardiopexy, considering gastric fundus anatomical peculiarities as an anti-reflux barrier.

**Methods:**

A retrospective analysis of pediatric patients presenting with refractory GERD from 2021 to 2023 was conducted. Inclusion criteria focused on primary GERD cases; secondary and redo cases were excluded. Diagnostic modalities included upper gastrointestinal contrast series and endoscopy. Demographic, operative, and postoperative data were assessed.

**Results:**

Among 57 cases, 8 with recurrent hiatal hernia were excluded. Enrolled cases (*n* = 49) had a mean age of 3.78 years and mean weight of 11.9 kg. All underwent laparoscopic ECCO procedure, with a mean operative time of 87 min.

During follow-up, six children experienced transient gas-bloat, and four had temporary dysphagia to solids. Two cases required revisions for absolute failures, while three managed partial recurrences with proton pump inhibitors. Of the total 49 cases, only nine required postoperative endoscopic assessment, which revealed a fully competent cardia with adequate wrapping in four of them. The remaining 40 cases demonstrated clinical improvement with the cessation of PPIs over a mean follow-up period of 11.6 months.

**Conclusions:**

‘Eversion Cruroplasty’ preserves crural pillar muscle excursion, avoiding segmentation seen with traditional suturing. The ‘Collar Overwrap’ achieves a 90% success rate, aligning the GE-junction while maintaining fundic pouch geometry, emphasizing its effectiveness and anatomical fidelity.

While surgery is frequently considered as a treatment option for medically refractory gastroesophageal reflux disease (GERD) [1, 2], deciding which surgical technique to opt to might be challenging. A variety of technical concerns may affect the success of anti-reflux surgery (ARS). In the pediatric population, these concerns may include whether to do a partial or total fundoplication, whether to divide the short gastric vessels or not, and whether to utilize a minimal or maximal dissection [3]. Noteworthy, with the goal of optimizing outcomes, the Nissen-Hill hybrid technique has recently been evolved in adult’s literature to combine the structural qualities of the two repairs, effectively balancing the shortcomings of each with each other’s strength; that is a full (360°) Nissen’s wrap maintains the radial integrity while Hill’s sutures secure the axial integrity [4].

However, the presenting author has lately advocated against wrapping the gastroesophageal junction (GEJ) with the gastric fundus, asserting that the dome-shaped gastric fundus itself works as a natural anti-reflux barrier in terms of the fundus receptive and adaptive relaxations as well as its substantially compressible dome-shaped fundal air bubble. Accordingly, the normal anatomical configuration or geometry of the gastric fundus should ideally not be distorted when creating a tension-free surgical wrap; that is to avoid the repercussions of the antral contraction waves exerting tensions onto the wrapped fundus around the GEJ [5]. Additionally, within the context of a continually growing body of a child, even a seemingly loosely applied suture cruroplasty today may pose a risk of strangulation in the future. Consequently, a cruroplasty technique is employed that does not impose strangulation on the evolving crural muscle pillars.

Thereby, the goal of this study is to introduce a novel hybrid approach, the Eversion Cruroplasty and Collar Overwrap (ECCO) procedure, which could include the desirable effects of the conventional partial wrapping, as well as the posteromedial cardiopexy, while considering the pointed out anatomo-physiological peculiarities of the gastric fundus.

## Methods

This study constitutes a retrospective analysis of consecutively performed laparoscopic ECCO procedures conducted at the authors’ institution between the years 2021 and 2023. The inclusion criteria encompassed children referred from the Pediatric Gastro-Enterology Unit presenting with refractory GERD, with or without concurrent hiatal hernia. The diagnostic modalities employed for all enrolled cases included upper gastrointestinal (GI) contrast series and endoscopy, facilitating the evaluation of the distal esophagus, competence of the cardia, hiatal herniation, Hill grading of the lower esophageal sphincter (LES), and the exclusion of instances featuring mechanical obstructions of the gastric outlet (i.e., secondary GERD).

Moreover, the analysis excluded redo cases, ensuring a focused examination of primary laparoscopic ECCO outcomes. Ethical approval for this study was obtained from the Institutional Review Board at Tanta University.

Relevant demographic information, including age at intervention, primary complaints, existence of neuro-logical impairment (NI), and medication history, is systematically documented. Detailed operative information, along with subsequent postoperative outcomes, is thoroughly evaluated.

## Surgical procedure

### Patient positioning and port-site placement

**Fig. 1:**
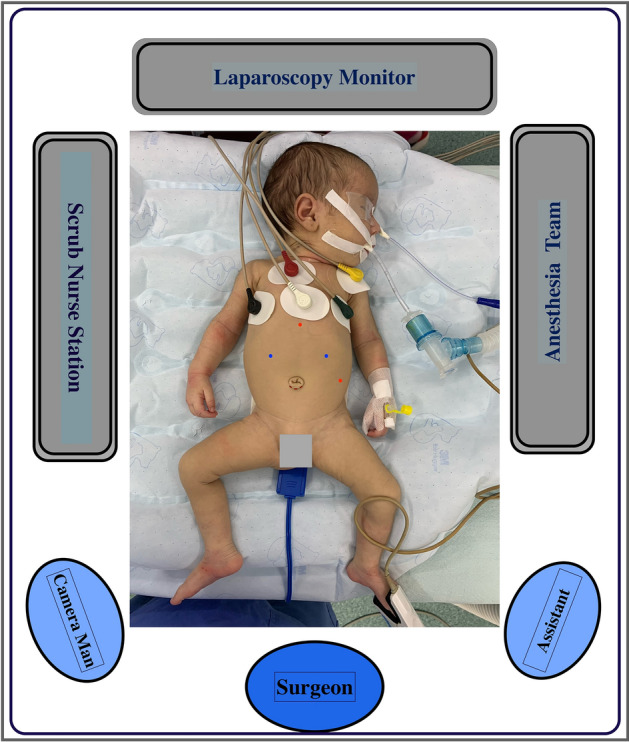
Diagram illustrating the operative room arrangement, patient positioning and port-site placement. A 5mm umbilical port is established for the placement of a 30° scope. Two 5 mm working instruments are introduced through ports or stab incisions of similar size, located at the linea semilunaris between the umbilical plane and costal margin on both sides (blue dots). An assisting retraction grasper is placed at the left anterior axillary line at the umbilical plane, and a 5 mm blunt grasper is introduced sub-xiphisternal for liver retraction (red dots) (Color figure online)

Following the administration of general endotracheal anesthesia, a soft orogastric tube is introduced to maintain gastric deflation. The patient is positioned supine in a slight anti-Trendelenburg’s orientation, with flexed knees resting on soft pads at the end of the operating table. Figure 1 illustrates the operative room arrangement, patient positioning and port-site placement.

### Gastroesophageal mobilization and mediastinal dissection

The division of the gastrohepatic ligament commences at the pars flacida above the caudate lobe of the liver, progressing towards the right crus, which is meticulously dissected from the esophagus while identifying the posterior vagus nerve.

Subsequently, the phrenoesophageal membrane is incised using a hook electrocautery, with simultaneous counter traction on the GEJ. The dissection begins at the right crus, circumferentially traversing the hiatus until reaching the dorsal confluence of the right and left limbs of the crus (the crural decussation). The gastrophrenic ligament is dissected to release the cardial notch, while preserving the anatomical recline of the gastric fundus to maintain the pyloro-dome gastric axis centered towards the dome of the left diaphragmatic copula.

Thereafter, electrocautery dissection is employed to free the lowermost mediastinal esophagus, until at least 2 cm of the esophagus is positioned within the abdomen, without exerting axial tension.

### Eversion cruroplasty (Fig. 2a and Fig. 3)

**Fig. 2:**
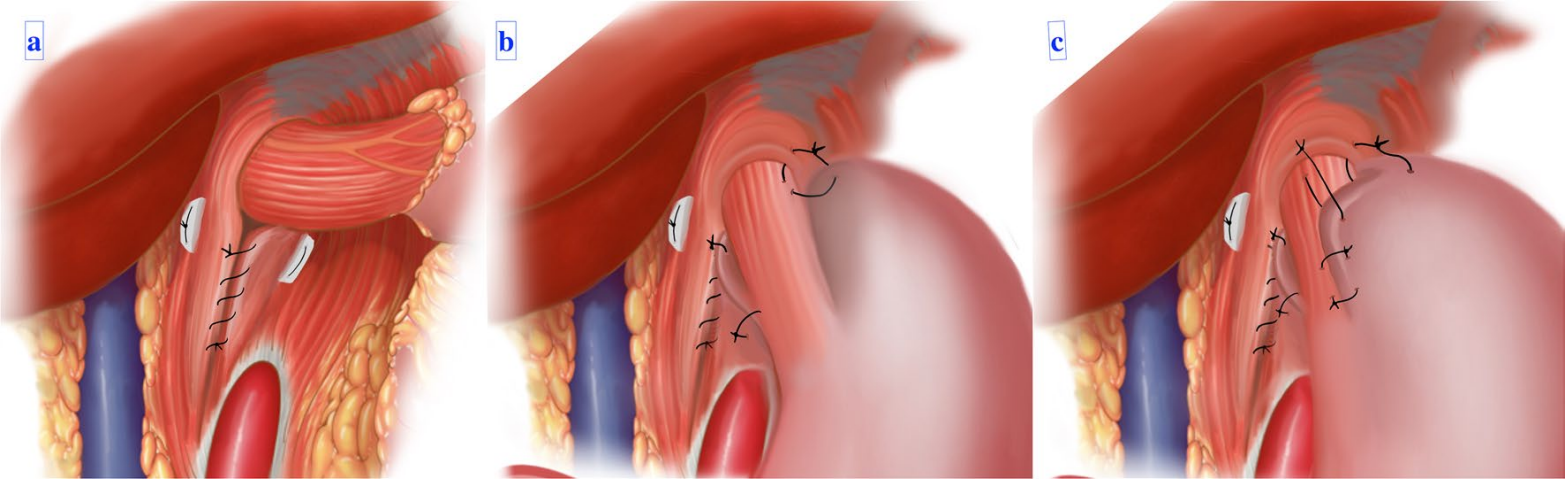
Diagram illustrating the proposed approach: **a** Eversion cruroplasty with crural apposition and eversion, using a running suture for fascia-to-fascia closure; **b**, **c** Demonstration of the precise placement of the proposed ‘collar overwrap’ sutures

**Fig. 3:**
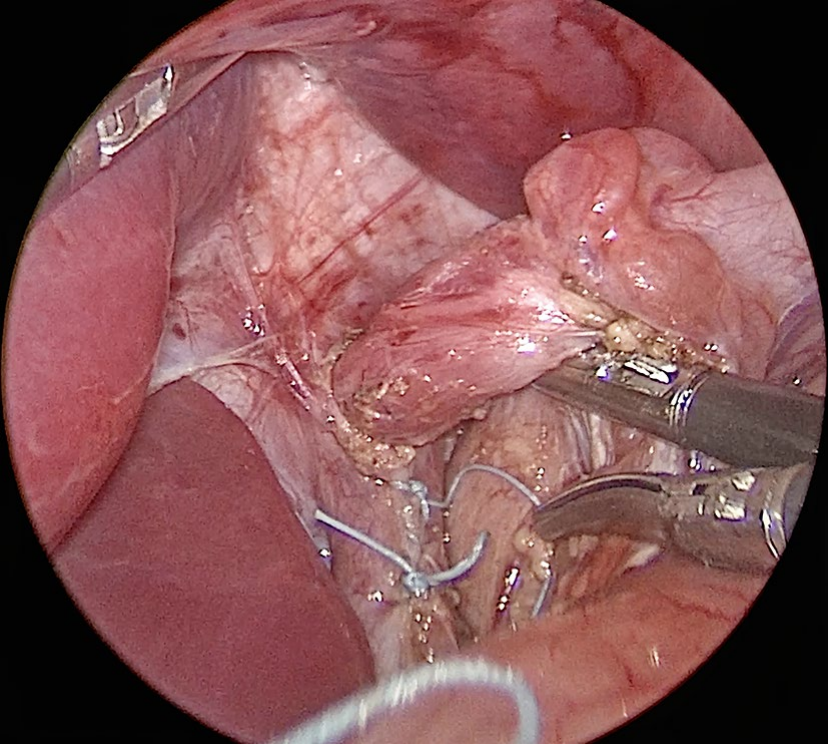
Operative photo demonstrating the ‘eversion cruroplasty’, which avoids the segmentation typically seen with the conventional through-and-through hiatal closure

Eversion cruroplasty is designed to maintain the efficacy of crural pillar muscle fiber contraction by avoiding the muscle segmentation effect associated with conventional deep-through-and-through suturing. Constant ventral and leftward traction is applied to the GEJ to expose the crural pillars adequately. A single transverse mattress suture, utilizing non-absorbable 2/0 braided Ethibond with PTFE pledgets, is deployed. This suture is positioned just lateral to the muscle pillars on both sides, specifically at the junction with the diaphragm, and subsequently tied to induce eversion of the crural pillars toward the abdominal domain. Next, a continuous closure of the everted crural pillars is performed, entailing a fascia-to-fascia suturing on the abdominal side.

### Assembly of the wrap (Fig. 2b,c and Fig. 4)

**Fig. 4:**
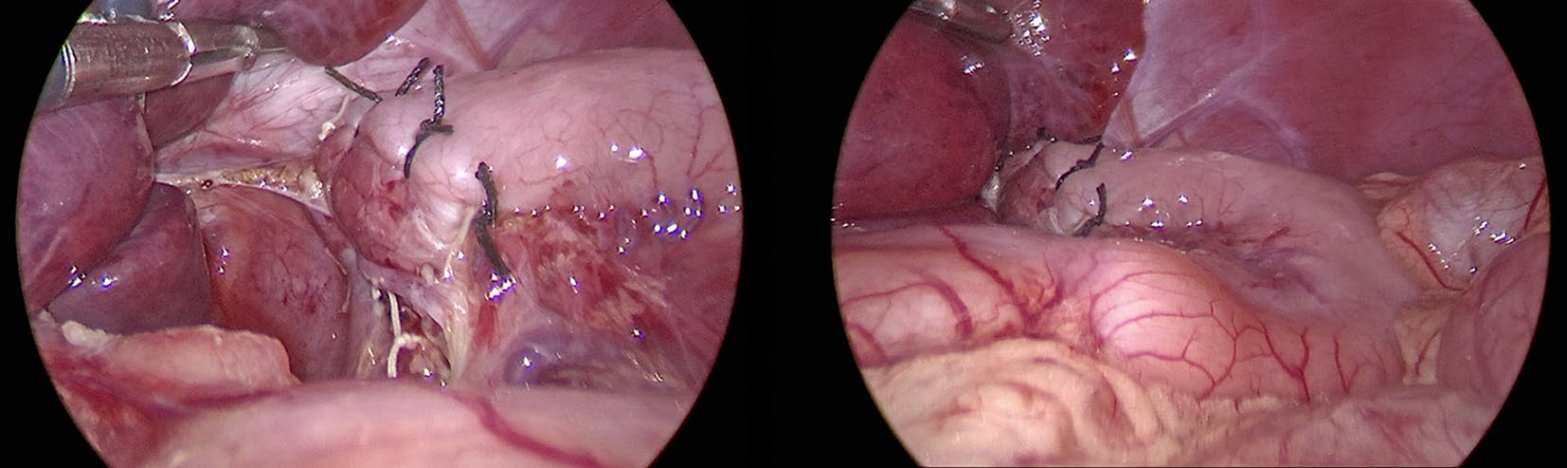
Operative photograph demonstrating the recline of the sided collar overwrap. The lower esophagus is nestled between the distensible gastric fundus on one side and the liver on the other, showcasing the spatial constraints

The construction of the adopted collar overwrap commences by securing the fundic curve, at 3 cm from the GEJ, to both the esophagus and the crural tendon at the 2 o’clock position. This initial stitch ensures that the fundic curve is not incorporated into the proposed wrap. Subsequently, a second stitch is applied to anchor the posteromedial portion of the GEJ to the pre-aortic fascia, constituting the caudal endpoint of the dorsal fold in the proposed collar overwrap.

The dorsal fold, primarily representing the adjacent bare area of the posterior wall of the stomach, is then radially anchored to the crural decussation (at 6 o’clock) and axially to the lower segment of the right pillar of the crus. Consequently, this fold serves to reinforce the hiatal cruroplasty, ensuring the containment of the wrapped LES within the abdominal cavity.

On the ventral side, the gastric fold—located between the anchored point of the fundic curve and the anteromedial point of the GEJ—forms the ventral fold of the proposed collar. This fold is wrapped over the distal esophagus and radially secured to the hiatus at the 12 o’clock position. Two to three additional axial sutures are then applied to tether this ventral fold of the collar wrap to the anterior esophageal wall at the 12 o’clock axis.

Thereby, a collar comprising the nearby gastric wall, containing sling fibers, encircles the distal esophagus, which contains the clasp fibers, in a 180° fashion (clockwise from 12 to 6 o’clock). This collar is dorsally affixed to the hiatus, right crus, and pre-aortic fascia, and ventrally anchored to the hiatus and abdominal esophagus.

Simultaneously, in cases involving neurological impairment (NI) or oropharyngeal dysfunctions posing a risk to oral feeding, gastrostomy tube placement is incorporated. All port sites are subsequently closed, and no abdominal drains are left in place.

### Postoperative care

On the second postoperative day, patients are initiated on oral fluid intake and maintained on this regimen for a duration of 5 days, followed by the gradual transition to a soft diet for the subsequent 2 weeks. Discharge occurs 48 h post-surgery for all cases. Proton pump inhibitors (PPIs) and gastroprokinetics are systematically discontinued within the period of 2 to 4 weeks following the procedure.

### Statistical analysis

Categorical variables are presented as counts and percentages, while continuous data are expressed as medians along with their corresponding ranges.

## Results

During the inclusion period of this study, 57 cases of refractory primary GERD were admitted to the authors’ facility. Of these, 8 cases with recurrent hiatal hernia at presentation were excluded from the analysis to enhance the homogeneity of the study group. The leading complaint in the presented series was persistent GERD symptoms (one or more symptom per case), despite adequate trials of conservative measures as stated in the referral form of the Gastro-Intestinal Unit. Table 1 provides an overview of the cohort’s demographics and the distribution of leading complaints. Table 2 summarizes the key objective findings from the diagnostic workup and operative exploration, as well as an overview of the outcomes.

**Table 1:** Overview of the cohort’s demographics and the distribution of leading complaints

Characteristic	Value
Total cases	49
Gender	
Male	32 (65.3%)
Female	17 (34.7%)
Mean age (years)	3.78 (Range: 0.67–18; Median: 2.5; IQR: 3.67)
Mean weight (Kg)	11.9 (Range; 4.4–45; Median: 10; IQR: 8.5)
Neurological impairment (NI)	8 (16.3%)
Leading complaints	
Persistent vomiting	28 (57.1%)
Failure to thrive	10 (20.4%)
Solid dysphagia	10 (20.4%)
Aspiration pneumonitis/PICU admissions	9 (18.4%)
Food aversion	6 (12.2%)
Retrosternal/epigastric pains	5 (10.2%)
Bronchial asthma	4 (8.2%)
Hematemesis	3 (6.1%)
Life-threatening event	1 (2.0%)
Arching backward (Sandifer’s syndrome)	1 (2.0%)
Feeding intolerance	1 (2.0%)
Persistent otitis media	1 (2.0%)

**Table 2:** Key objective findings from the diagnostic workup and operative exploration, as well as an overview of the outcomes

Diagnostic Findings	Count (*n*)	Percentage (%)	Outcomes	Count (*n*)	Percentage (%)
Sliding Hiatal Hernia (SHH)	28	57.1%	Transient Gas-Bloat	6	12.2%
Markedly Incompetent Cardia (No SHH)	7	14.3%	Temporary Dysphagia to Solid Foods		
Type IV Hiatal Hernia (Cascade Stomach)	2	4.1%	New-Onset Dysphagia Cases	4	8.2%
Peptic Stricture	3	6.1%	Pre-op Dysphagia Cases	6	12.2%
Post-Esophageal Atresia Repair	1	2.0%	Need for Post-operative Endoscopic Assessment	9	18.4%
Congenital Esophageal Stricture	1	2.0%	Absolute Failure	2	4.1%
Unsafe Oral Feeds (Oropharyngeal Dyskinesia)	4	8.2%	Partial Recurrence of GERD Symptoms	3	6.1%
Ladd’s Band	6	12.2%	Persistent Failure to Thrive	1	2.0%
Sub-Hepatic Cecum	4	8.2%	Post-operative Cessation of PPIs	40	81.6%
Gastroparesis	2	4.1%			

All cases underwent laparoscopic ECCO procedure as technically described. The mean operative time was 87 min (Range: 70–105 min; Median: 85; IQR: 25). Eight NI cases—four of which had a preoperative diagnosis of severe sensory impairment leading to unsafe oral feeds—received supplementary feeding gastrostomy tube placement by the end of the procedure.

No conversions to open surgery occurred, and there were no significant blood losses necessitating blood transfusions. However, three cases exhibited preoperative hematemesis and required blood transfusions. With the exception of one NI case with Pickwickian syndrome*, no cases required postoperative admission to the Pediatric Intensive Care Unit (PICU).

*(Obesity with central apnea, hypothyroidism, and severe autistic features, tracheostomized with home mechanical ventilation).

During the follow-up, six children experienced transient gas-bloat during the initial 2–6 weeks after the operation. Among them, two cases with concurrent indwelling feeding gastrostomy were recommended to intermittently release the gas-bloat through the gastrostomy button.

Four cases exhibited temporary new-onset postoperative dysphagia to solid foods, showing spontaneous improvement within 2–6 weeks. Among those with a concurrent preoperative diagnosis of solid dysphagia (*n* = 6), a lengthier duration for the resolution of dysphagia was reported (Range: 2–12 weeks). In cases with esophageal stricture, postoperative balloon dilation was scheduled in three sessions, spaced one month apart, along with prolonged use of PPIs and mucosal protectives throughout the dilation regimen.

Within the studied series, two cases experienced absolute failures, indicating recurrences that necessitated revisions. These instances occurred after an initial clinical improvement of GERD symptoms, specifically at 4 and 9 months post-surgical repair. It is noteworthy that both cases exhibited persistent violent postoperative retching. Upper GI series and EGD-scopy confirmed recurrent sliding hiatal hernia (SHH) with wrap failure and an incompetent cardia in both cases. Upon redo laparoscopic exploration, a complete failure of the crural repair and the wrap’s posterior leaflet fixation was revealed. Hence, redo laparoscopic ECCO procedure were successfully performed for both cases. Following a follow-up period of 9 and 11 months, both cases demonstrated abstinence from medical treatment. Additionally, three other cases reported controlled partial recurrence of GERD symptoms, effectively managed by PPIs.

**Fig. 5:**
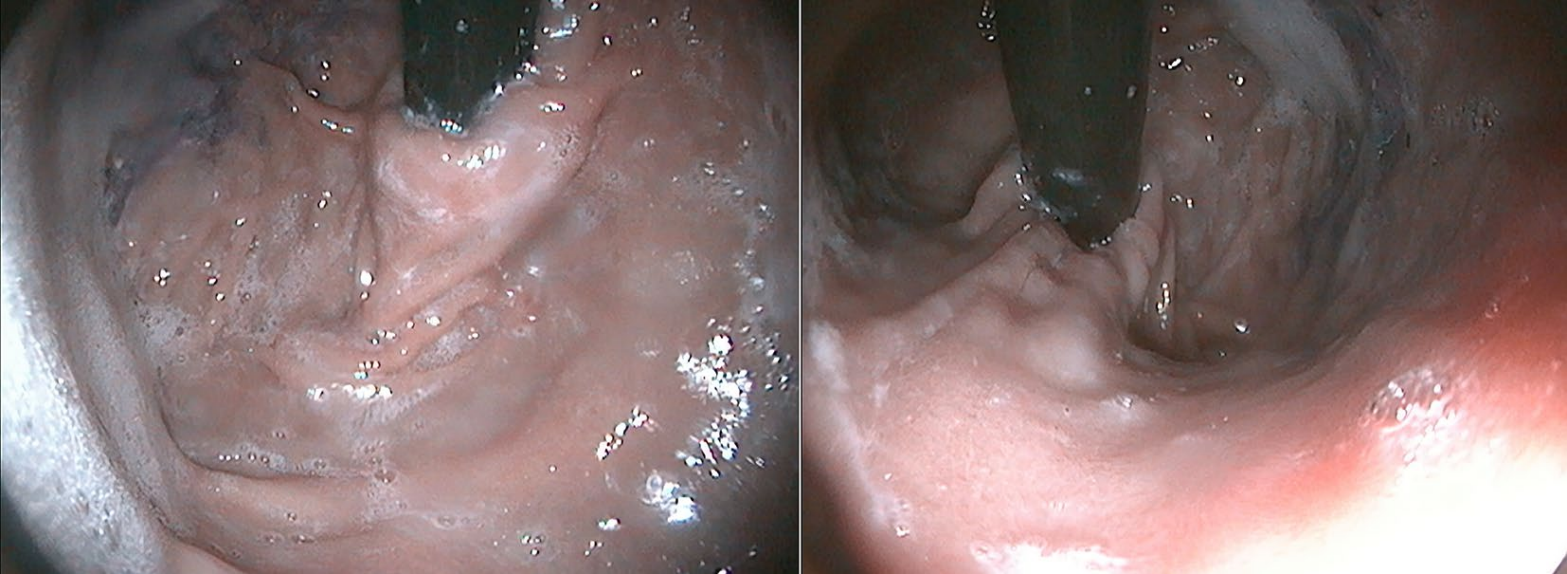
Follow-up endoscopic retroversion view of an operated case demonstrating the adequate gripping around the scope and the intact geometry of the gastric fundus

Nine cases underwent postoperative endoscopic assessment led by persistent failure to thrive (*n* = 1), a dilation regimen for esophageal stricture (*n* = 3), and documented recurrence of GERD symptoms (*n* = 5). Except for the five cases with failure, all exhibited a competent cardia, with adequate wrapping, a good length of abdominal esophagus, and a preserved angle between the pyloro-dome axis and esophageal axis (Fig. 5). The case with persistent failure to thrive was diagnosed with feeding intolerance due to pylorospasm, for which a pyloric balloon dilation was done.

The remaining cases (*n* = 40) showed clinical improvement in their primary GERD symptoms during the follow-up at the outpatient clinic, with a mean follow-up period of 11.6 months (Range: 1–32 months; Median: 8.5; IQR: 13). This improvement was manifested by the cessation of PPIs and gastroprokinetics, serving as a good negative predictor for the failure of anti-reflux surgery. It is noteworthy that three of these cases reported sporadic vomiting episodes only during acute illnesses; gastroenteritis (*n* = 1) and bronchopneumonia (*n* = 2).

## Discussion

The known complex anatomic geometry that goes into the reflux barrier and that needs to be addressed during any surgical procedure includes: the crural diaphragm or hiatal canal, the LES, especially its overall and intra-abdominal length, and the musculomucosal flap valve controlled by the angle of His. All work in concert to constitute a functioning anti-reflux barrier [6–8]. At GE-endoscopy, this valve can be graded according to Hill into four grades [9]; that is with progressive effacement or shortening of the valve length, there is a correlative loss of the valve function. This has been shown to be more predictive of the severity of GERD than LES pressure, likely because the Hill grading reflects the sum of all components of anti-reflux barrier rather than simply measuring LES pressure [6, 10].

Surgical fundoplication remains a mainstay in the stepwise management of the disease, as it addresses the underlying incompetence of the GE valve rather than merely reducing the acid production [11–13]. The two basic types being performed are the ‘total wrap,’ which includes the Nissen fundoplication, and the ‘partial wrap,’ which includes the Thal and Toupet fundoplications. Selecting the best technique, though, is still debatable [14].

In children, partial fundoplication resulted in more favorable outcomes compared to total fundoplication, including reductions in long-term dysphagia, the need for upper GI endoscopy with or without dilation, and postoperative PPIs use by 2.2%, 9.3%, and 3.2%, respectively. However, the only undesirable outcome associated with partial fundoplication was a 10% increase in wrap failures [3]. Moreover, promising results of the Hill-Snow repair in children have been documented [15]. As a central tenet, performing the right operation correctly on a given patient requires a thorough understanding of the underlying pathophysiology and the anatomic and physiologic components of the reflux barrier [16].

Considering additional components working to support the anti-reflux barrier is essential in the context of anti-reflux surgery. The gastrointestinal tract, from mouth to anus, generally exhibits a continuous tubular configuration, deviating only in the instances of the stomach and cecum. In these latter structures, the organoaxial alignment exclusively assumes an angulated disposition relative to the axis of their respective preceding segments. Such an alignment might operate as a physical barrier akin to valves, precluding an otherwise free ‘orad’ flow of luminal contents into their preceding segments. Failure to do so would have deleterious backwash consequences on the distal esophagus and the terminal ileum, respectively [5].

Further, the anatomical configuration and geometry of the stomach enable it to play a crucial role in handling the laminar flow pattern of digesta within its parts, contributing significantly to the anti-reflux mechanism [17]. The gastric fundus, characterized by a lack of myenteric interstitial cells of Cajal, is predominantly electrically quiescent. This feature allows for gastric accommodation reflexes, facilitating proximal stomach expansion without a substantial increase in intragastric pressure—a mechanism supporting temporary food storage [18–22]. Impaired gastric accommodation is often linked to disorders such as GERD and dyspepsia [17]. The normal muscle tone of the proximal stomach is restored during gastric emptying, maintaining the common cavity pressure levels [23]. Accordingly, alterations in gastric geometry can markedly impact gastric biomechanics, influencing gastric flow, motility, and emptying [24, 25].

Bouras used single photon emission computed tomography to examine gastric volumes in patients who underwent previous fundoplication [26]. The study revealed a notable decrease in postprandial adaptive relaxation of the proximal stomach following Nissen fundoplication, despite preserved receptive relaxation. This abnormality is suggested to play a role in the development of frequently reported dyspeptic symptoms, as supported by another study using an intragastric barostatic balloon [27].

However, the specific contributions to this abnormal postprandial pattern—whether it stems from fundal detachment and denervation, the wrap itself, or the preoperative GERD—are still unclear. Additionally, in patients with prior Nissen fundoplication, there is a significantly faster return of intragastric volume to preprandial levels, likely resulting in the swift emptying of liquids from the stomach, as frequently documented in the literature [28–30].

In the same context of understanding how gastric geometry impacts biomechanics, literature identifies four types of the fundal gastric bubble: the hemispherical dome type, irregular type, stomach type (outlining the stomach), and undetected type. The type of fundal bubble serves as an informative marker for assessing upper digestive tract functional disorders, including decreased LES pressure, delayed gastric emptying, or impaired proximal stomach accommodation. Individuals with GERD symptoms are most frequently associated with the stomach-type bubble, followed by undetected-type, irregular-type, and unlikely with the dome-type [31]. Moreover, it has been reported that the air–liquid interface within the dome-shaped fundus plays a crucial role in maintaining gastric mucosa homeostasis [32]. These findings underscore the importance of preserving normal gastric geometry, particularly that of the fundus, during anti-reflux surgery.

Further, the diverse modes of presentation of GERD in children, as documented in the literature and observed within the studied cohort, may underscore the imperative to tailor surgical approaches to address the specific needs of such a growing anatomy to mitigate surgery-related pathophysiology. Consequently, this study, aligning with the Nissen-Hill Hybrid technique in the adult’s literature [4], implements a hybrid approach, utilizing a partial wrap, along with its attendant favorable outcomes in children, complemented by Hill’s gastropexy to fortify the axial integrity of the repair.

From another perspective, given the established efficacy of the crura as an anti-reflux mechanism [33–35], there is prevalent advocacy among surgeons for its closure [36]. However, the conventional approach of employing robust, through-and-through muscle sutures—often utilizing non-absorbable materials—suitable for adult cruroplasty may not be appropriate for the continually developing anatomy of a child. In a growing body, even a seemingly loosely applied suture today may pose a risk of strangulation in the future. Therefore, the proposed ‘Eversion Cruroplasty’ is designed to safeguard the muscle excursion of the crural pillars by mitigating the segmentation effect induced by traditional suturing. Additionally, it maintains the mass contractility and sliding properties of the muscle fibers within their fascial compartments.

Overall, in the pursuit of biomimicry, this study addresses the imperative objective of restoring the anatomical recline of the GEJ and preserving the muscle excursion of the crural pillars during anti-reflux surgery. Hence, a nuanced approach ‘ECCO’ is adopted through a sided partial wrapping over the esophagus, involving the bloated gastric fundus working against the right crus and caudate lobe of the liver. This dynamic interplay is meticulously controlled by the pars condensa of the gastrohepatic ligament in the normal anatomy. Such multifaceted interventions collectively contribute to a comprehensive strategy for addressing the functional dynamics within the GE region.

The reported 90% success rate in the presented series, and the follow-up endoscopic evaluations proved the ability to reproduce an augmented anatomical alignment of the GE-junction while preserving the geometry of the fundic pouch. The preserved capacity for belching may itself serve to safeguard the surgical repair by functioning as a pop-off valve, counteracting increments in the common cavity pressure. It is noteworthy that the cohort under consideration exhibited a restored ability to belch within a period of 2–6 weeks postoperatively, with some cases (*n* = 3) reported episodes of emesis exclusively during systemic illness, similar to their non-refluxing peers. Additionally, the two reported recurrences in the enrolled series were associated with a well-manifested violent retching behavior in the postoperative period.

The study’s limitations include the need for more comprehensive research with long-term follow-up, particularly involving a control group of children who have undergone more standard procedures, such as Nissen or Hill-Snow procedures.

## Conclusions

The diverse presentations of pediatric GERD suggest unique surgical needs for this population. The proposed ‘Eversion Cruroplasty’ preserves the muscle excursion of the evolving crural pillars by mitigating the segmentation effect of traditional suturing. With a reported 90% success rate, the ‘Collar Overwrap’ achieves anatomical alignment of the GE-junction while preserving fundic pouch geometry, as opposed to the conventional wrapping of the fundus around the esophagus. This underscores the effectiveness and anatomical fidelity of the ECCO procedure.
